# A case of cellular variants of Sclerosing epithelioid fibrosarcoma resembling Plasmacytoma/Myeloma: Diagnostic difficulty in the fine needle aspiration

**DOI:** 10.1016/j.ijscr.2020.03.012

**Published:** 2020-03-07

**Authors:** Nahoko Nagano, Noriyoshi Ishikawa, Mamiko Nagase, Asuka Araki, Teruaki Iwahashi, Riruke Maruyama

**Affiliations:** a Department of Surgical Pathology, Shimane University Hospital, Japan; b Department of Organ Pathology, Shimane University School of Medicine, Japan

**Keywords:** Fine needle aspiration biopsy, Sclerosing epithelioid sarcoma, MUC4, EWSR1, Low-grade fibromyxoid sarcoma, Case report

## Abstract

**Introduction:**

Sclerosing epithelioid sarcoma (SEF) is a rare fibroblastic sarcoma. It is classically composed of cords of epithelioid cells embedded in a hyalinized stroma; however, cases of cellular variants also exist. A cellular variants of SEF can mimic Plasmacytoma/Myeloma (PM) and myoepithelioma. Hence, accurate diagnosis of SEF is important for cytologists and pathologists.

**Presentation of case:**

We present the case of a 75-year-old female patient diagnosed with a cellular variant of SEF occurring in the erector spinal muscle. Immunostaining of MUC4 and fluorescence in situ hybridization of EWSR1 (break-apart signal) were used for diagnosis.

**Conclusion:**

The cellular variants of SEF presented diagnostic difficulties in fine needle aspiration. Moreover, it could not be distinguished from PM.

## Highlights

• It is difficult to diagnose cellular variants of Sclerosing epithelioid sarcoma by fine needle aspiration.
• If dense areas of tumor cells are collected by FNA, multiple myeloma and myoepithelioma can be different diagnosis.
• MUC4 is a very useful immunological marker for diagnosis of Sclerosing epithelioid sarcoma.

## 1 Introduction

Sclerosing epithelioid sarcoma (SEF) is a rare soft tissue tumor characterized by epithelioid fibroblasts arranged in distinct cords and nests in a densely sclerotic, hyalinized stroma [1]. SEF is difficult to diagnose because it can mimic metastatic carcinoma, hematological neoplasms such as lymphoma, multiple myeloma, and mesenchymal neoplasms such as myoepithelioma [2]. As a high-density variant, a case of “non-fibrosing SEF” has also been reported [2]. A subset of SEF appears to be related to low-grade fibromyxoid sarcoma (LGFS) [3]. Both tumors are usually positive for MUC4 in immunostaining [4] and several cases of hybrid SEF and LGFS have been reported [[5], [6], [7], [8]]. The major fusion gene of SEF is EWSR1-CREB3L1 [9]; however, the FUS-CREB3L2 fusion gene has also been reported, which is the characteristic fusion gene of LGFS [5].

We describe a cytological and pathological case of SEF including recent genetic insights. We encountered cellular variants of SEF in fine needle aspiration (FNA). Differential diagnosis with Plasmacytoma/Myeloma (PM) was required in this case. This research work has been reported in line with SCARE 2018 criteria [10].

## 2 Case presentation

**Fig. 1:**
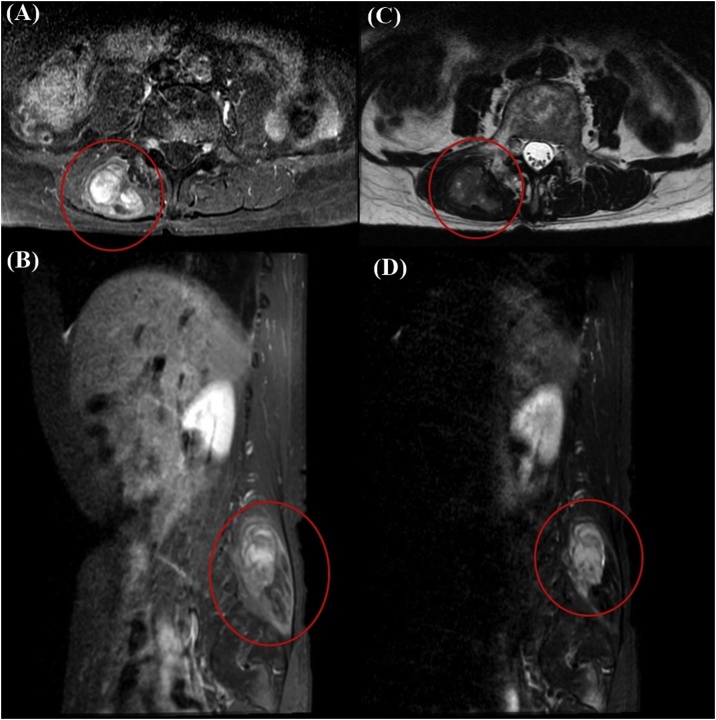
MRI image of the tumor. Horizontal section (A). Sagittal section (B) of T1-contrast WIs. Horizontal section of T2 WIs (C). Sagittal section of fat suppression T2 WIs (D).

A 75-year-old female underwent a detailed examination for pancytopenia at her previous medicine. Contrast CT revealed a 5 × 3 × 3.3 cm tumor with imaging effect on the internal side of the mass in the right erector spinal muscle. The patient was referred to the orthopedic department in Shimane university hospital for further examination. MRI revealed high density in the T1-contrast weighted images (WIs) (Fig. 1A, B) and iso and multifocal high density in the T2-WIs (Fig. 1C). In the fat suppression T2-WIs, high-density area was recognized inside the tumor (Fig. 1D). FNA was performed under ultrasound guidance and a malignant tumor was detected. Subsequently, tumor resection was performed. As for pancytopenia, it was the diagnosis of myelodysplastic syndrome by the subsequent bone marrow examination.

### 2.1 Cytology of the FNA specimens (Fig. 2)

Most of the tumor cells are isolated and composed of eccentrically located nuclei and an oxyphilic cytoplasm. Nuclei have fine chromatin and unclear nucleoli. Abundant cytoplasm somewhat resembles Golgi apparatus. Sometimes binuclear tumor cells were observed.

**Fig. 2:**
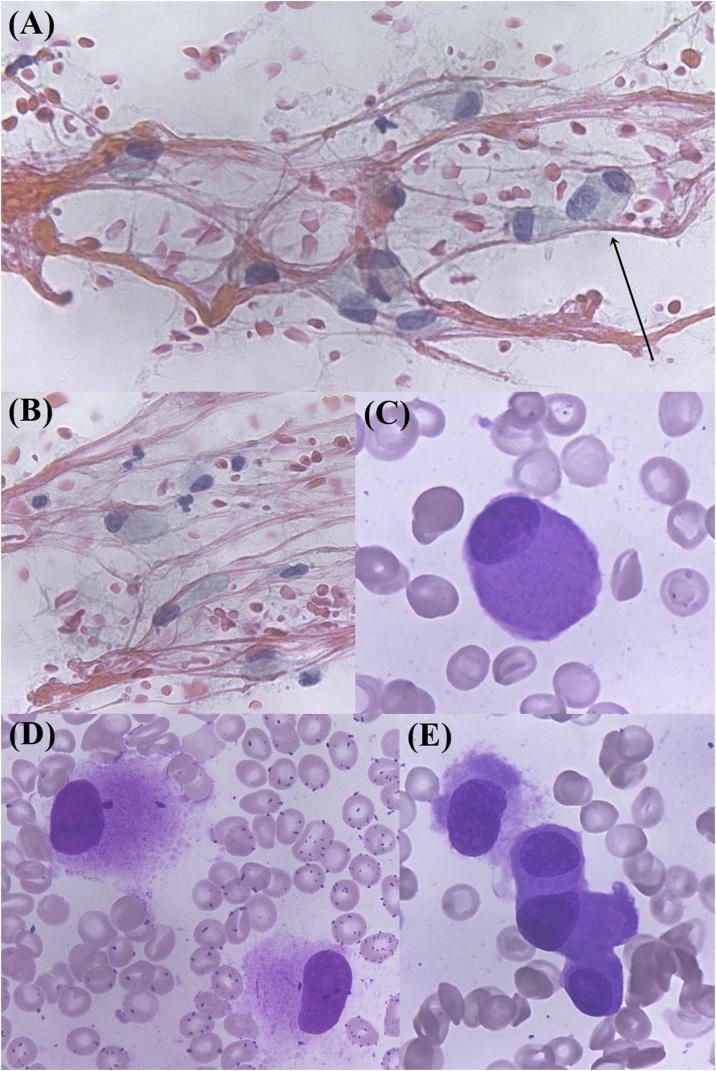
Cytological image of the FNA specimen.Papanicolaou staining (A, B). Giemsa staining (C, D, E). Poorly cohesive neoplastic cells were observed. These cells are composed of abundant eosinophilic cytoplasm; nuclei have fine chromatin and located at the periphery of the cytoplasm; nucleoli are unclear. Sometimes binuclear cells are observed (arrows).

### 2.2 Histology of the FNA specimens

**Fig. 3:**
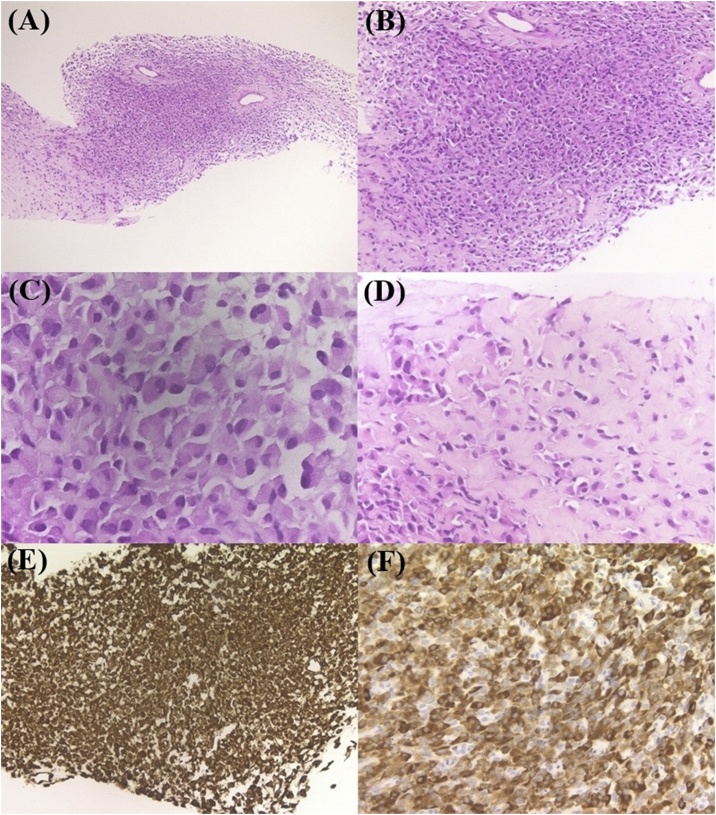
Histological image of FNA specimen.Different density areas of neoplastic cells are shown (A). High-density area of the tumor (B). Neoplastic cells have eccentric and eosinophilic cytoplasm (C). Mitosis is hardly observed (C). Neoplastic cells are embedded in the sclerotic stroma in the low-density area (D). Neoplastic cells are diffusely positive for vimentin (E) and MUC4 (F).

There are regions of different density of tumor cells (Fig. 3A). The tumor cells have an eccentric and eosinophilic cytoplasm (Fig. 3B), with no increase in mitosis (Fig. 3C). In low-density area, abundant and weakly eosinophilic stroma can be seen (Fig. 3D). Immunohistochemistry revealed tumor cells that were diffusely positive for vimentin (Fig. 3E) and MUC4 (Fig. 3F), focally and weekly positive for CD99, and negative for AE1/AE3, CAM5.2, Myeloperoxidase, CD138, LCA, Myogenin, Desmin, αSMA, MyoD1, CD31, CD34, WT-1, ERG, D2-40, MDM2, c-kit, S100, HMB45, Melan A, and Mib-1 index is 3%.

### 2.3 Macroscopic and microscopic findings of surgical resected specimens

**Fig. 4:**
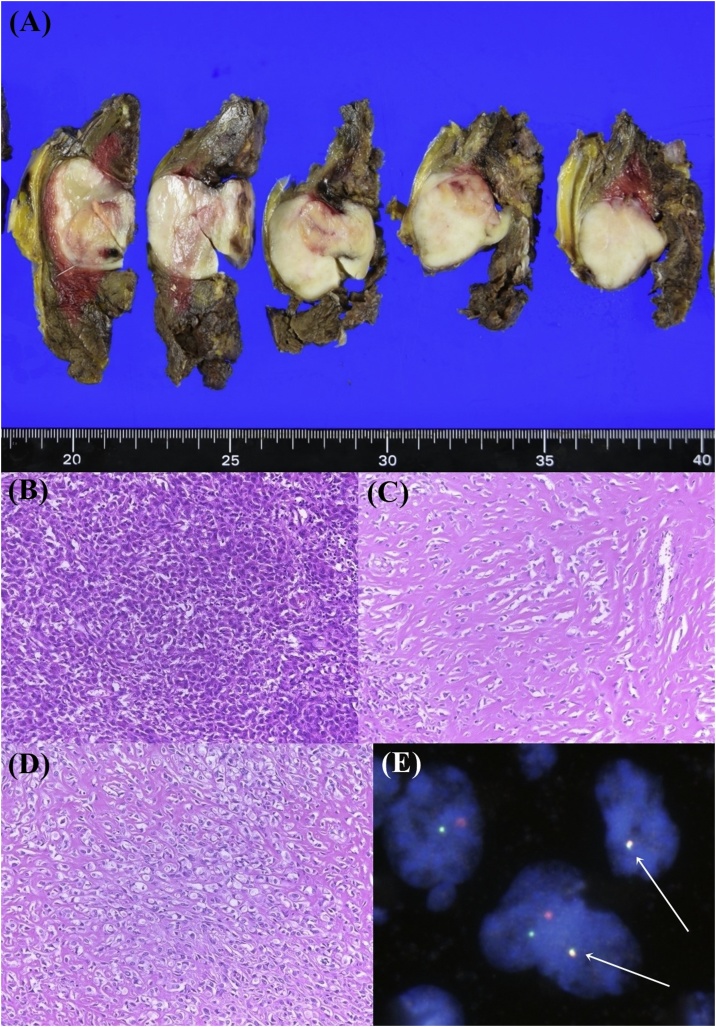
Macroscopic, microscopic and FISH analyses of surgical specimens. Tumor surface is white and boundary is well-defined (A). High- (B) and low- (C) density area of neoplastic cells. The latter area is sclerotic. Epithelioid feature of neoplastic cells (D). Break-apart signal of EWSR1 (E). The red labeled probes were situated proximal to EWSR1 and, the green labeled probes were situated distal to EWSR1. Arrows indicate fusion genes.

The surface color of the tumor was white and the boundary between the surrounding muscle and the tumor surface was relatively well-defined despite absence of a tumor capsule (Fig. 4A). Even in the surgical specimen, regions with non-sclerotic high density of tumor cells (Fig. 4B) and sclerotic low density of tumor cells (Fig. 4C) were observed. In the Sclerosing area, corded growth of epithelioid cells within a densely sclerotic stroma, which is a typical feature of SEF, was also observed (Fig. 4D).

### 2.4 Fluorescence in situ hybridization (FISH) analysis

EWSR1 break-assay FISH (EWSR1 Breakapart kit, Cytocell Ltd, Cambridge), and EWSR1-CREB3L1, EWSR1-CREB3L2 fusion FISH (EWSR1:RP11-305J10, CREB3L1:RP11-1106J11, CREB3L2: RP-11-377B19) were performed on interphase nuclei from paraffin-embedded sections.

We detected break-apart signals of EWSR1 (Fig. 4E) but, could not detect fusion signals of EWSR1-CREB3L1 or EWSR1-CREB3L2 (data not shown). We also performed FUS-CREB3L1, FUS-CREB3L2 fusion assays (FUS: RP11-157F22), neither of these fusion genes was detected.

## 3 Discussion

SEF is a malignant fibroblastic neoplasm, which occurs in middle-aged and elderly patients [1]. It occurs in the deep-seated region, most commonly affecting the lower extremity [1]. The typical histological feature of this tumor is presence of epithelioid fibroblasts [1] arranged in cords or nests in a densely sclerotic, hyalinized stroma [1].

SEF has a differential diagnosis that includes metastatic tumor such as epithelial carcinoma, hematologic disease [2] such as PM, and other mesenchymal tumors such as myoepithelioma [2]. It requires special attention when a region with high-density of tumor cells is collected by FNA, as presented in this case. In this case of FNA specimen, discohesive tumor cells with eccentrically located nuclei and relatively eosinophilic abundant cytoplasm were identified. Therefore, the possibility of myoepithelioma and PM had become a differential diagnosis. In addition to these findings, presence of Golgi apparatus-like appearance and a few binuclear tumor cells were also observed. Hence, it was initially considered to be PM.

However, typical cells of PM exhibit irregular shape, chromatin aggregation, and nucleolus is generally absent [11]. Tumor cells in our study did not completely meet these criteria. But, cellular findings of PM vary from case to case [11], some with less chromatin aggregation in the nucleus, others with less nuclear irregularities or unclear perinuclear halo. We concluded that it is difficult to determine the histological diagnosis only from the morphological findings. Furthermore, we should have distinguished it from PM, when the Dutcher body or Russel body was found to be absent [11].

If the tumor in this case was PM, we hypothesized that faintly eosinophilic, amorphous area in a small range observed in the biopsy material was amyloid. However, we could not detect amyloid deposits by DFS staining. In the regions with low density of tumor cells with faintly eosinophilic stroma, we hypothesized it to be SEF because tumor cells were embedded with a cord-like structure. To confirm this, immunostaining with MUC4 was performed. MUC4 is a very useful immunological marker in differential diagnosis of LGFS and SEF. This case was strongly and diffusely positive for MUC4, which confirmed the diagnosis of SEF.

SEF and LGFS are considered to be related to each other [3], and several cases of hybrid tumors having the histology of both SEF and LFMS have been reported [[5], [6], [7], [8]]. Typical SEF cases harboring the EWSR1-CREB3L1 fusion gene have been described in the past [9,12]. However, cases with the FUS-CREB3L2 fusion gene, which is a characteristic of LGFS, have also been reported [5]. In this case, EWSR1-CREB3L1 and FUS-CREB3L2 fusion genes were not detected in FISH. Therefore, we searched for EWSR1 genes using break-apart probe. As a result, the break-apart signals of EWSR1 were detected. There are some reports of EWSR1-CREB3L2 fusion genes in SEF [3,5]; however, in this case the EWSR1-CREB3L2 fusion gene [13,14] could not be detected by FISH. In addition, EWSR1-CREB3L3 and other fusion genes (e.g., FUS-CREM, PAX5-CREB3L1) have also been reported in SEF [5]. However, these reports warrant further research.

As in this case, if SEF had a region with different density of tumor cells and high-density area is collected by FNA for diagnostic purposes, it may fall into an unexpected pitfall. When encountered with a non-typical histology of SEF, as in this case, it is necessary to conduct immunostaining with MUC4 to confirm SEF in the case of a slightly uncomfortable feeling in FNA specimen.

## Declaration of Competing Interest

None.

## Sources of funding

None.

## Ethical approval

Ethical approval has been exempted by my institution for reporting this case.

## Consent

Written informed consent was obtained from the patient for publication of this case report and accompanying images.

## Author contribution

Nagano N and Ishikawa N: conception of reporting case, data recording, and drafting.

Nagano N, Ishikawa N, Araki A and Iwahashi T: Management of case.

Nagase M, Maruyama R: Critical revision of article and final approval of the version to be submitted.

## Registration of research studies

Registration number is researchregistry5387.

## Guarantor

Ishikawa N.

## Provenance and peer review

Not commissioned, externally peer-reviewed.
